# Pattern and Clinical Significance of CA19‐9 Expression in Human Cancer: A Tissue Microarray Study on 14,966 Tumors

**DOI:** 10.1002/cam4.71710

**Published:** 2026-03-12

**Authors:** Nina Schraps, Anne Menz, Florian Viehweger, Seyma Büyücek, David Dum, Ria Schlichter, Andrea Hinsch, Christoph Fraune, Christian Bernreuther, Martina Kluth, Claudia Hube‐Magg, Katharina Möller, Viktor Reiswich, Andreas M. Luebke, Patrick Lebok, Sören Weidemann, Guido Sauter, Maximilian Lennartz, Frank Jacobsen, Till S. Clauditz, Andreas H. Marx, Ronald Simon, Stefan Steurer, Baris Mercanoglu, Nathaniel Melling, Thilo Hackert, Eike Burandt, Natalia Gorbokon, Sarah Minner, Till Krech, Florian Lutz, Morton Freytag

**Affiliations:** ^1^ General, Visceral and Thoracic Surgery Department and Clinic University Medical Center Hamburg‐Eppendorf Hamburg Germany; ^2^ Institute of Pathology University Medical Center Hamburg‐Eppendorf Hamburg Germany; ^3^ Institute of Pathology Clinical Center Osnabrueck Osnabrueck Germany; ^4^ Department of Pathology Academic Hospital Fuerth Fuerth Germany

**Keywords:** biomarker, CA19‐9, cancer, carbohydrate antigen 19–9, IHC, tissue microarray

## Abstract

**Background:**

Carbohydrate antigen 19–9 (CA19‐9) is a cell surface glycoprotein widely used as a diagnostic and prognostic serum marker for monitoring pancreatic cancer. The aim of this study was to evaluate the prevalence and clinical relevance of CA19‐9 expression in human cancer.

**Methods:**

To comprehensively determine the prevalence and clinical relevance of CA19‐9 expression in cancer, a tissue microarray containing 14,966 samples from 134 different tumor types and subtypes as well as 608 samples of 76 different normal tissue types was analyzed by immunohistochemistry.

**Results:**

The staining results were categorized into four groups according to the percentage of CA19‐9‐positive tumor cells and the staining intensity. At least an occasional CA19‐9 positivity was found in 79 of 134 tumor categories with 66 containing at least one strongly positive case. CA19‐9 positivity was most frequent in biliopancreatic adenocarcinomas (84.4%–95.4%), adenocarcinomas of the upper and lower gastrointestinal tract (54.7%–74.4%), embryonal carcinomas of the testis (93.5%), and endometrioid endometrial carcinomas (87.5%). High CA19‐9 staining was associated with advanced pT‐stage, pN1, L1 (*p* < 0.0001 each), V1 (*p* = 0.0239), mismatch repair protein deficiency (*p* = 0.0067), and BRAF V600E mutation (*p* = 0.0005) in colorectal adenocarcinoma. Reduced CA19‐9 staining was associated with distant metastasis (*p* = 0.0067) in clear cell renal cell carcinoma, advanced pT‐stage (*p* = 0.0277) in papillary renal cell carcinoma, high grade (*p* = 0.0129) in breast cancer and with invasive tumor growth (pTa vs. pT2‐4, *p* < 0.0001) and high grade (*p* = 0.0367) in urothelial carcinoma.

**Conclusions:**

It is concluded that a high CA19‐9 expression, as found in pancreatic adenocarcinoma, occurs in subsets of various other cancer types. Patients with such tumors could potentially benefit from CA19‐9 serum monitoring, for example in terms of response to treatment, recurrence or predicting prognosis. Cancer type dependent associations between high or low CA19‐9 expression and aggressive tumor phenotype are in line with the complex and variable functional role described for CA19‐9 in cancer.

## 1 Introduction

Carbohydrate antigen 19–9 (CA19‐9), also known as sialyl‐Lewis A, is a cell surface glycoprotein with a role in cell‐to‐cell recognition [1]. CA19‐9 is a tetrasaccharide carbohydrate consisting of a transmembrane protein skeleton and several extracellular oligosaccharide chains which are highly glycosylated [1, 2]. Because the Lewis gene product, 1,4‐fucosyltransferase is needed for the formation of CA19‐9, expression of this glycoprotein complex is only possible in individuals with Le (a‐b+) or Le (a + b‐) blood groups [2, 3, 4, 5]. Because CA19‐9 is often highly overexpressed in pancreatic cancers, serum measurement of CA19‐9 has earlier been recommended for early detection of this cancer [1, 6, 7]. However, as elevated CA19‐9 serum levels can also be found in a variety of benign conditions, this approach has been abandoned but CA19‐9 serum measurement is still used as a marker for disease monitoring in pancreatic cancer and other gastrointestinal cancers [1, 6, 7, 8].

More than 3500 studies have measured serological CA19‐9 in cancer, and at least 145 studies have investigated CA19‐9 tissue expression by immunohistochemistry (IHC). Overall, these findings have indicated that CA19‐9 expression is common in pancreatic, billiary, gastric, esophageal, colorectal cancer and ovarian cancer but that many other cancer types can also show CA19‐9 expression, occasionally even at high levels. Data on the prevalence of CA 19–9 expression in specific tumor entities vary considerably in the literature, however. For example, reported CA19‐9 positivity rates obtained by IHC ranged from 25.8% to 100% in gastric cancer [9, 10], 41.6%–100% in colorectal cancer [9, 11], 30.0%–72.5% in adenocarcinoma of the lung [12, 13], 33.3%–100% in mucinous carcinoma of the ovary [14, 15], 7.0%–68.7% in serous carcinoma of the ovary [14, 16], and 53.8%–100% in ductal adenocarcinoma of the pancreas [17, 18]. Technical factors, such as staining protocols and antibodies used, different definitions of thresholds to determine positivity, as well as possible selection bias with respect to the analyzed tumors may have caused these discrepancies.

To better understand and systematically assess the relative importance of CA19‐9 expression in different cancer types and normal tissues, more than 14,900 tissue samples from 134 different tumor types and subtypes, and 76 non‐neoplastic tissues were evaluated by IHC in a tissue microarray (TMA) format.

## 2 Material and Methods

Tissue Microarrays (TMAs). The normal tissue TMA consisted of 8 samples from 8 different donors for each of the 76 different normal tissue types (608 samples on one slide). A total of 14,966 primary tumors from 134 tumor types and subtypes were contained in the cancer TMAs. Detailed histopathological data on grade, pathological tumor stage (pT), pathological lymph node status (pN), blood vessel infiltration (V), or lymph vessel infiltration were available from 598 pancreatic cancers, 2351 colon cancers, 327 gastric cancers, 518 thyroid cancers, 1224 clear cell and 310 papillary renal cell carcinomas (RCC), 369 serous and 40 endometrial ovarian cancers, 182 endometrioid endometrial carcinomas, 600 invasive breast cancers of no special type (NST), and 829 urothelial carcinomas. A detailed description of the composition of both normal and cancer TMAs can be found in the results section. All samples were from the archives of the Institutes of Pathology, University Medical Center Hamburg‐Eppendorf, Germany, the Institute of Pathology, Clinical Center Osnabrueck, Germany, and Department of Pathology, Academic Hospital Fuerth, Germany. Tissues were fixed in 4% buffered formalin and embedded in paraffin. The TMA manufacturing process has previously been described [19, 20]. In brief, one tissue spot (diameter: 0.6 mm) per patient was used. A prerequisite for the TMA construction was a tumor diameter of at least 1 cm on the tumor‐containing block. The use of archived remnants of diagnostic tissues for manufacturing of TMAs and their analysis for research purposes, as well as patient data analysis, has been approved by local laws (HmbKHG, §12) and by the local ethics committee (Ethics commission Hamburg, WF‐049/09). All work has been carried out in compliance with the Helsinki Declaration.

### 2.1 Immunohistochemistry (IHC)

Freshly cut TMA sections were immunostained on 1 day and in one experiment. Slides were deparaffinized with xylol, rehydrated through a graded alcohol series, and exposed to heat‐induced antigen retrieval for 5 min in an autoclave at 121°C in pH 7.8 Tris‐EDTA‐Citrat (TEC) puffer. Endogenous peroxidase activity was blocked with Dako REAL Peroxidase‐Blocking Solution (Agilent Technologies, Santa Clara, CA, USA; #S2023) for 10 min. Primary antibody specific against CA19‐9 protein (mouse monoclonal, HMV333, ardoci GmbH, Hamburg, Germany) was applied at 37°C for 60 min at a dilution of 1:150. Using the Dako REAL EnVision Detection System Peroxidase/DAB+, Mouse kit (Agilent Technologies, Santa Clara, CA, USA; #K5007), the bound antibody was visualized (according to the manufacturer's directions). For validation purposes, the normal tissue TMA was analyzed by the mouse animal monoclonal CA19‐9 antibody clone 1116‐NS‐19‐9 (Agilent Technologies, Santa Clara, CA, USA). The protocol was as follows. Freshly prepared 2.5 μm TMA sections were applied in a Dako Omnis automated stainer (Agilent Technologies, Santa Clara, CA, USA) using the EnVision FLEX, High pH Kit (Agilent Technologies, Santa Clara, CA, USA, #GV800). Slides were deparaffinized with Clearify agent (Agilent Technologies, Santa Clara, CA, USA, #GC810) and exposed to heat‐induced antigen retrieval for 30 min at 97°C in target retrieval solution, high pH reagent (part of Agilent kit #GV800). Primary antibody specific for CA19‐9 (1116‐NS‐19‐9, Agilent Technologies, Santa Clara, CA, USA) was applied at ambient temperature for 20 min at a dilution of 1:100. Endogenous peroxidase activity was blocked with peroxidase blocking‐reagent (part of Agilent kit #GV800) for 3 min. The bound antibody was visualized using the EnVision FLEX, High pH kit reagents DAB+ Chromogen and Substrate Buffer (parts of Agilent kit #GV800) and EnVision FLEX + Mouse LINKER (Agilent Technologies, Santa Clara, CA, USA; #GV821) according to the manufacturer's directions. Hemalaun was used as a counterstain. For tumor tissues, the percentage of CA19‐9 positive tumor cells was estimated, and the staining intensity was semi‐quantitatively recorded (0, 1+, 2+, 3+) [21]. The staining results were categorized into four groups: Negative: no staining at all, weak staining: staining intensity of 1+ in ≤ 70% or staining intensity of 2+ in ≤ 30% of tumor cells, moderate staining: staining intensity of 1+ in > 70%, staining intensity of 2+ in > 30% but in ≤ 70% or staining intensity of 3+ in ≤ 30% of tumor cells, strong staining: staining intensity of 2+ in > 70% or staining intensity of 3+ in > 30% of tumor cells.

### 2.2 Statistics

Statistical calculations were performed with JMP18 software (SAS, Cary, NC, USA). Contingency tables and the chi [2]‐test were performed to search for associations between CA19‐9 staining and tumor phenotype. A pairwise comparison was not performed if there was a clear trend within a group.

## 3 Results

### 3.1 Technical Issues

A total of 12,403 (82.9%) of 14,966 tumor samples were interpretable in our TMA analysis. Non‐interpretable samples demonstrated lack of unequivocal tumor cells or lack of entire tissue spots. At least 4 samples of each normal tissue type were evaluable on our normal tissue TMA.

### 3.2 CA19‐9 Immunostaining in Normal Tissues

**FIGURE 1:**
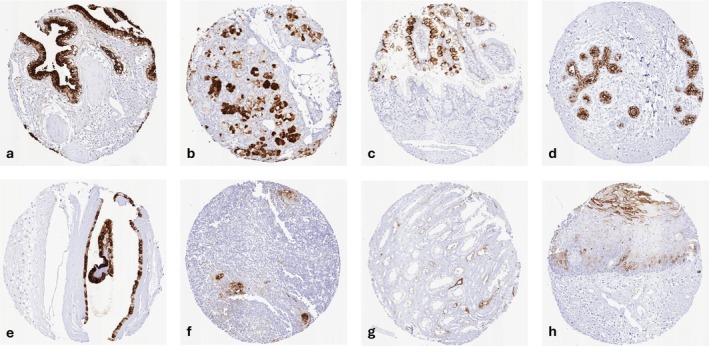
CA19‐9 immunostaining of normal tissues. The panels show strong CA19‐9 immunostaining in epithelial cells of the gallbladder (a), sublingual gland (b), ileal villae (c), the breast (d), and in a large subset of amnion cells of the placenta (e). A less intense CA19‐9 staining was observed in corpuscles of Hassall's of the thymus (f), and a small subset of collecting duct cells in the renal medulla (g), and a subset of squamous epithelial cells in a sample from the oral cavity (h).

A strong CA19‐9 staining was seen in a subset of surface epithelial cells (especially goblet cells) of the intestine, gastric glandular cells, gallbladder epithelium, intrahepatic bile ducts, subsets of glandular cells (mostly mucinous) in salivary and bronchial glands, luminal breast epithelial cells, endocervical epithelium, subsets of respiratory epithelial cells (mostly goblet cells), and in amnion cells. A less intense CA19‐9 staining was observed in corpuscles of Hassall's of the thymus, individual cells or groups of cells in squamous epithelia, few tubular and collecting duct cells of the kidney, few cells of the cauda epididymis, few prostatic epithelial cells, few endometrial cells, epithelial cells of the fallopian tube, and a fraction of urothelial cells, especially in the superficial cell layer. CA19‐9 was always absent in mesenchymal and hematolymphoid tissues, brain, pituitary gland, thyroid, adrenal gland, parathyroid, testis, placenta, lung, seminal vesicle, hepatocytes, and the ovary. It is of note that complete absence of CA19‐9 staining occasionally occurred in all tissues. This is in line with an estimated 10% of analyzed samples being derived from Le a‐b‐ patients [22, 23, 24]. Representative images are shown in (Figure 1). A similar staining pattern was observed in all cell types when the anti‐CA‐19 antibody 1116‐NS‐19‐9 was used although staining was somewhat weaker (Figure S1).

### 3.3 CA19‐9 Immunostaining in Neoplastic Tissues

**FIGURE 2:**
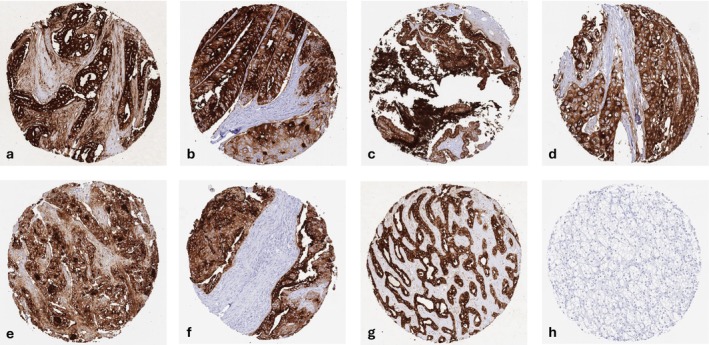
CA19‐9 immunostaining in cancer. The panels show strong CA19‐9 positivity in cancer cells of an adenocarcinoma of the pancreas (a), a colorectal adenocarcinoma (b), a high‐grade serous carcinoma of the ovary (c), an invasive urothelial carcinoma (d), a breast carcinoma of no special type (e), a gastric adenocarcinoma (f), and in a cholangiocarcinoma of the liver (g). CA19‐9 staining is absent in a clear cell renal cell carcinoma (h).

**FIGURE 3:**
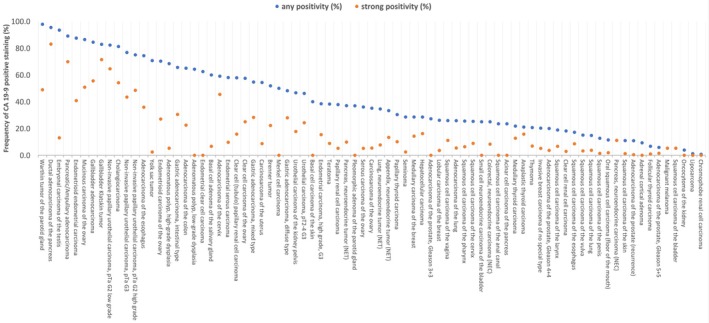
Ranking order of CA19‐9 positive immunostaining in different human tumors. Orange dots show the percentage of strongly stained samples, while blue dots show the percentage of positive samples of any intensity.

A positive CA19‐9 immunostaining was observed in 4740 (38.2%) of 12,403 analyzable tumors, including 2199 (17.7%) with weak, 612 (4.9%) with moderate, and 1929 (15.5%) with strong staining intensity. The staining pattern was predominantly membranous but also cytoplasmic. Representative images are shown in (Figure 2). At least an occasional weak CA19‐9 positivity was detected in 79 of 134 tumor categories and 66 entities included at least one case with strong CA19‐9 positivity (Table 1). CA19‐9 positivity was most frequently seen in Warthin tumor of the parotid gland (97.9%), biliopancreatic adenocarcinomas (84.4%–95.4%), adenocarcinomas of the upper and lower gastrointestinal tract (54.7%–74.4%), as well as in a few tumors of the male and female genital tract including embryonal carcinoma of the testis (93.5%), endometrioid endometrial carcinoma (87.5%), and mucinous carcinoma of the ovary (86.4%). CA19‐9 was less common in squamous cell carcinoma (5.3%–25.9%) and absent in tumors of the hematopoietic and lymphoid tissue as well as most mesenchymal tumors. A graphical representation of a ranking order of CA19‐9 positive and strongly positive cancers is given in (Figure 3). Associations with tumor phenotype are summarized in (Table 2). A high CA19‐9 expression level was associated with advanced pT stage, nodal metastasis, lymphovascular infiltration (*p* < 0.0001 each), blood vessel invasion (*p* = 0.0239), mismatch repair deficiency (*p* = 0.0067), and BRAF V600E mutation (*p* = 0.0005) in colorectal adenocarcinomas. Most of these associations were also retained in mismatch repair proficient tumors (Table S1). CA19‐9 positivity was also linked to nodal metastasis in breast cancer (*p* = 0.0269) and urothelial carcinoma (*p* = 0.0212). Reduced CA19‐9 staining was associated with distant metastasis (*p* = 0.0067) in clear cell RCC, advanced pT stage (*p* = 0.0277) in papillary RCC, and higher grade in breast cancer (*p* = 0.0129) as well with invasive tumor growth (pTa vs. pT2‐4, *p* < 0.0001) and high grade in invasive urothelial carcinoma. CA19‐9 immunostaining was unrelated to parameters of tumor aggressiveness in endometrial, ovarian, pancreatic and thyroidal cancer.

**TABLE 1:** CA19‐9 Immunostaining in human tumors.

	Tumor entity	CA19‐9 immunostaining					
		TMA (*n*)	Analyzable (*n*)	Negative (%)	Weak (%)	Moderate (%)	Strong (%)
Tumors of the skin	Basal cell carcinoma of the skin	41	20	60.0	30.0	10.0	0.0
	Squamous cell carcinoma of the skin	95	82	89.0	8.5	1.2	1.2
	Malignant melanoma	19	19	94.7	0.0	0.0	5.3
	Malignant melanoma lymph node metastasis	86	62	100.0	0.0	0.0	0.0
	Merkel cell carcinoma	2	2	50.0	50.0	0.0	0.0
Tumors of the head and neck	Squamous cell carcinoma of the larynx	109	90	81.1	10.0	2.2	6.7
	Squamous cell carcinoma of the pharynx	60	47	74.5	17.0	2.1	6.4
	Oral squamous cell carcinoma (floor of the mouth)	130	105	88.6	4.8	4.8	1.9
	Pleomorphic adenoma of the parotid gland	50	46	63.0	34.8	2.2	0.0
	Warthin tumor of the parotid gland	49	47	2.1	27.7	21.3	48.9
	Basal cell adenoma of the salivary gland	15	15	40.0	46.7	6.7	6.7
Tumors of the lung, pleura and thymus	Adenocarcinoma of the lung	196	147	74.1	15.6	4.8	5.4
	Squamous cell carcinoma of the lung	80	54	85.2	9.3	1.9	3.7
	Mesothelioma, epithelioid	40	26	100.0	0.0	0.0	0.0
	Mesothelioma, biphasic	29	20	100.0	0.0	0.0	0.0
	Thymoma	29	29	79.3	10.3	3.4	6.9
	Lung, neuroendocrine tumor (NET)	29	26	65.4	11.5	15.4	7.7
Tumors of the female genital tract	Squamous cell carcinoma of the vagina	30	27	74.1	11.1	3.7	11.1
	Squamous cell carcinoma of the vulva	107	93	84.9	8.6	3.2	3.2
	Squamous cell carcinoma of the cervix	88	79	74.7	12.7	3.8	8.9
	Adenocarcinoma of the cervix	23	22	40.9	4.5	9.1	45.5
	Endometrioid endometrial carcinoma	288	272	12.5	39.3	7.4	40.8
	Endometrial serous carcinoma	36	31	41.9	48.4	0.0	9.7
	Carcinosarcoma of the uterus	57	46	45.7	43.5	2.2	8.7
	Endometrial carcinoma, high grade, G3	13	13	61.5	15.4	7.7	15.4
	Endometrial clear cell carcinoma	9	8	37.5	50.0	12.5	0.0
	Endometrioid carcinoma of the ovary	93	74	29.7	32.4	10.8	27.0
	Serous carcinoma of the ovary	530	444	64.0	25.7	5.2	5.2
	Mucinous carcinoma of the ovary	75	59	13.6	28.8	6.8	50.8
	Clear cell carcinoma of the ovary	51	40	42.5	22.5	10.0	25.0
	Carcinosarcoma of the ovary	47	37	64.9	27.0	2.7	5.4
	Granulosa cell tumor of the ovary	44	30	100.0	0.0	0.0	0.0
	Leydig cell tumor of the ovary	4	2	100.0	0.0	0.0	0.0
	Sertoli cell tumor of the ovary	1	1	100.0	0.0	0.0	0.0
	Sertoli Leydig cell tumor of the ovary	3	3	100.0	0.0	0.0	0.0
	Steroid cell tumor of the ovary	3	3	100.0	0.0	0.0	0.0
	Brenner tumor	32	27	48.1	14.8	14.8	22.2
Tumors of the breast	Invasive breast carcinoma of no special type	499	390	79.7	10.3	4.9	5.1
	Lobular carcinoma of the breast	150	111	73.9	19.8	2.7	3.6
	Medullary carcinoma of the breast	8	7	71.4	14.3	0.0	14.3
	Tubular carcinoma of the breast	2	1	100.0	0.0	0.0	0.0
	Mucinous carcinoma of the breast	7	6	100.0	0.0	0.0	0.0
Tumors of the digestive system	Adenomatous polyp, low‐grade dysplasia	50	28	35.7	53.6	10.7	0.0
	Adenomatous polyp, high‐grade dysplasia	50	38	31.6	55.3	7.9	5.3
	Adenocarcinoma of the colon	2483	2278	34.9	33.4	9.2	22.5
	Gastric adenocarcinoma, diffuse type	215	168	51.8	13.1	7.1	28.0
	Gastric adenocarcinoma, intestinal type	215	154	34.4	27.9	7.1	30.5
	Gastric adenocarcinoma, mixed type	62	53	45.3	22.6	3.8	28.3
	Adenocarcinoma of the esophagus	83	39	25.6	28.2	10.3	35.9
	Squamous cell carcinoma of the esophagus	76	35	82.9	8.6	0.0	8.6
	Squamous cell carcinoma of the anal canal	91	68	76.5	16.2	7.4	0.0
	Cholangiocarcinoma	58	48	18.8	20.8	6.3	54.2
	Gallbladder adenocarcinoma	51	45	15.6	20.0	8.9	55.6
	Gallbladder Klatskin tumor	42	35	17.1	8.6	2.9	71.4
	Hepatocellular carcinoma	312	284	71.5	10.6	1.8	16.2
	Ductal adenocarcinoma of the pancreas	659	483	4.6	7.0	5.4	83.0
	Pancreatic/Ampullary adenocarcinoma	98	73	11.0	13.7	5.5	69.9
	Acinar cell carcinoma of the pancreas	18	17	76.5	23.5	0.0	0.0
	Gastrointestinal stromal tumor (GIST)	62	61	100.0	0.0	0.0	0.0
	Appendix, neuroendocrine tumor (NET)	25	15	66.7	20.0	0.0	13.3
	Colorectal, neuroendocrine tumor (NET)	12	8	100.0	0.0	0.0	0.0
	Ileum, neuroendocrine tumor (NET)	53	46	100.0	0.0	0.0	0.0
	Pancreas, neuroendocrine tumor (NET)	101	81	63.0	18.5	8.6	9.9
	Colorectal, neuroendocrine carcinoma (NEC)	14	12	75.0	16.7	0.0	8.3
	Ileum, neuroendocrine carcinoma (NEC)	8	7	100.0	0.0	0.0	0.0
	Gallbladder, neuroendocrine carcinoma (NEC)	4	4	100.0	0.0	0.0	0.0
	Pancreas, neuroendocrine carcinoma (NEC)	14	9	88.9	0.0	0.0	11.1
Tumors of the urinary system	Non‐invasive papillary urothelial carcinoma, pTa G2 low grade	87	79	17.7	12.7	5.1	64.6
	Non‐invasive papillary urothelial carcinoma, pTa G2 high grade	80	68	25.0	16.2	10.3	48.5
	Non‐invasive papillary urothelial carcinoma, pTa G3	126	99	23.2	21.2	12.1	43.4
	Urothelial carcinoma, pT2‐4 G3	735	552	53.8	15.4	6.5	24.3
	Squamous cell carcinoma of the bladder	22	19	94.7	0.0	0.0	5.3
	Small cell neuroendocrine carcinoma of the bladder	5	4	75.0	0.0	25.0	0.0
	Sarcomatoid urothelial carcinoma	25	14	100.0	0.0	0.0	0.0
	Urothelial carcinoma of the kidney pelvis	62	45	53.3	22.2	6.7	17.8
	Clear cell renal cell carcinoma	1287	1097	81.9	12.6	2.6	2.9
	Papillary renal cell carcinoma	368	288	62.2	26.4	6.3	5.2
	Clear cell (tubulo) papillary renal cell carcinoma	26	19	42.1	36.8	5.3	15.8
	Chromophobe renal cell carcinoma	170	141	99.3	0.7	0.0	0.0
	Oncocytoma of the kidney	257	181	96.1	3.3	0.6	0.0
Tumors of the male genital organs	Adenocarcinoma of the prostate, Gleason 3 + 3	83	55	72.7	25.5	1.8	0.0
	Adenocarcinoma of the prostate, Gleason 4 + 4	80	55	80.0	14.5	1.8	3.6
	Adenocarcinoma of the prostate, Gleason 5 + 5	85	68	94.1	4.4	0.0	1.5
	Adenocarcinoma of the prostate (recurrence)	258	239	89.1	9.2	1.3	0.4
	Small cell neuroendocrine carcinoma of the prostate	2	1	100.0	0.0	0.0	0.0
	Seminoma	682	563	71.4	22.4	3.7	2.5
	Embryonal carcinoma of the testis	54	46	6.5	63.0	17.4	13.0
	Leydig cell tumor of the testis	31	19	100.0	0.0	0.0	0.0
	Sertoli cell tumor of the testis	2	2	100.0	0.0	0.0	0.0
	Sex cord stromal tumor of the testis	1	1	100.0	0.0	0.0	0.0
	Spermatocytic tumor of the testis	1	1	100.0	0.0	0.0	0.0
	Yolk sac tumor	53	41	29.3	53.7	14.6	2.4
	Teratoma	53	34	61.8	23.5	5.9	8.8
	Squamous cell carcinoma of the penis	92	71	87.3	9.9	1.4	1.4
Tumors of endocrine organs	Adenoma of the thyroid gland	63	63	100.0	0.0	0.0	0.0
	Papillary thyroid carcinoma	341	326	69.6	13.5	6.7	10.1
	Follicular thyroid carcinoma	109	104	93.3	4.8	1.0	1.0
	Medullary thyroid carcinoma	57	55	78.2	7.3	1.8	12.7
	Parathyroid gland adenoma	43	22	100.0	0.0	0.0	0.0
	Anaplastic thyroid carcinoma	19	19	78.9	5.3	0.0	15.8
	Adrenal cortical adenoma	48	43	90.7	9.3	0.0	0.0
	Adrenal cortical carcinoma	27	24	100.0	0.0	0.0	0.0
	Pheochromocytoma	51	48	100.0	0.0	0.0	0.0
Tumors of hematopoetic and lymphoid tissues	Hodgkin's lymphoma	103	90	100.0	0.0	0.0	0.0
	Small lymphocytic lymphoma, B‐cell type (B‐SLL/B‐CLL)	50	44	100.0	0.0	0.0	0.0
	Diffuse large B cell lymphoma (DLBCL)	113	103	100.0	0.0	0.0	0.0
	Follicular lymphoma	88	81	100.0	0.0	0.0	0.0
	T‐cell non‐Hodgkin's lymphoma	25	22	100.0	0.0	0.0	0.0
	Mantle cell lymphoma	18	15	100.0	0.0	0.0	0.0
	Marginal zone lymphoma	16	13	100.0	0.0	0.0	0.0
	Diffuse large B‐cell lymphoma (DLBCL) in the testis	16	15	100.0	0.0	0.0	0.0
	Burkitt lymphoma	5	4	100.0	0.0	0.0	0.0
Tumors of soft tissue and bone	Granular cell tumor	23	14	100.0	0.0	0.0	0.0
	Leiomyoma	50	49	100.0	0.0	0.0	0.0
	Leiomyosarcoma	94	91	100.0	0.0	0.0	0.0
	Liposarcoma	96	86	98.8	1.2	0.0	0.0
	Malignant peripheral nerve sheath tumor (MPNST)	15	14	100.0	0.0	0.0	0.0
	Myofibrosarcoma	26	25	100.0	0.0	0.0	0.0
	Angiosarcoma	42	29	100.0	0.0	0.0	0.0
	Angiomyolipoma	91	72	100.0	0.0	0.0	0.0
	Dermatofibrosarcoma protuberans	21	13	100.0	0.0	0.0	0.0
	Ganglioneuroma	14	12	100.0	0.0	0.0	0.0
	Kaposi sarcoma	8	2	100.0	0.0	0.0	0.0
	Neurofibroma	117	86	100.0	0.0	0.0	0.0
	Sarcoma, not otherwise specified (NOS)	74	66	100.0	0.0	0.0	0.0
	Paraganglioma	41	32	100.0	0.0	0.0	0.0
	Ewing sarcoma	23	11	100.0	0.0	0.0	0.0
	Rhabdomyosarcoma	7	6	100.0	0.0	0.0	0.0
	Schwannoma	122	99	100.0	0.0	0.0	0.0
	Synovial sarcoma	12	9	100.0	0.0	0.0	0.0
	Osteosarcoma	19	11	100.0	0.0	0.0	0.0
	Chondrosarcoma	15	10	100.0	0.0	0.0	0.0
	Rhabdoid tumor	5	4	100.0	0.0	0.0	0.0
	Solitary fibrous tumor	17	17	100.0	0.0	0.0	0.0

**TABLE 2:** CA19‐9 immunostaining and tumor phenotype.

		*n*	CA19‐9 immunostaining				
			Negative (%)	Weak (%)	Moderate (%)	Strong (%)	*p*
**Adenocarcinoma of the colon**	**pT1**	86	36.0	43.0	11.6	9.3	< 0.0001
	**pT2**	427	39.1	35.8	10.3	14.8	
	**pT3**	1230	34.6	33.4	8.5	23.5	
	**pT4**	432	31.7	30.6	9.3	28.5	
	**pN0**	1132	39.5	33.6	8.7	18.3	< 0.0001
	**pN+**	1033	30.7	33.7	9.5	26.1	
	**V0**	1561	35.6	34.4	9.5	20.5	0.0239
	**V1**	568	32.9	32.2	8.1	26.8	
	**L0**	707	39.0	35.8	9.3	15.8	< 0.0001
	**L1**	1434	33.5	32.5	8.7	25.2	
	**left side**	1249	30.7	38.0	10.4	20.9	0.0131
	**right side**	470	32.6	31.5	8.9	27.0	
	**MMR proficient**	1167	30.7	38.3	11.2	19.8	0.0067
	**MMR deficient**	87	40.2	21.8	9.2	28.7	
	**RAS wildtype**	466	32.4	38.6	10.3	18.7	0.0683
	**RAS mutation**	359	26.7	37.3	10.0	25.9	
	**BRAF wildtype**	129	39.5	36.4	8.5	15.5	0.0005
	**BRAF V600E mutation**	22	18.2	18.2	4.5	59.1	
**Adenocarcinoma of the colon, MMR proficient**	**pT1**	47	34.0	44.7	10.6	10.6	0.042
	**pT2**	239	34.7	40.2	12.1	13.0	
	**pT3**	641	30.7	38.1	10.8	20.4	
	**pT4**	229	25.3	37.1	11.8	25.8	
	**pN0**	594	34.5	38.7	10.9	15.8	0.0021
	**pN+**	553	26.6	38.3	11.6	23.5	
	**V0**	826	31.1	39.6	11.4	17.9	0.3779
	**V+**	310	29.0	36.5	10.6	23.9	
	**L0**	467	34.9	40.7	10.3	14.1	0.0003
	**L1**	655	27.6	37.1	11.6	23.7	
	**left**	894	30.5	39.6	11.4	18.5	0.2433
	**right**	267	31.1	34.5	10.9	23.6	
	**RAS Mutation**	289	26.0	37.0	12.1	24.9	0.0022
	**wildtype**	361	33.0	43.5	8.9	14.7	
	**BRAF V600E**	9	22.2	22.2	0.0	55.6	0.0501
	**wildtype**	107	36.4	41.1	7.5	15.0	
**Endometrioid endometrial carcinoma**	**pT1**	107	11.2	39.3	12.1	37.4	0.0717
	**pT2**	24	8.3	54.2	0.0	37.5	
	**pT3‐4**	37	18.9	51.4	2.7	27.0	
	**pN0**	50	8.0	54.0	6.0	32.0	0.1948
	**pN+**	30	23.3	36.7	3.3	36.7	
**Clear cell renal cell carcinoma**	**ISUP**						
	**1**	257	78.6	16.3	4.3	0.8	0.9059
	**2**	391	77.2	16.4	5.1	1.3	
	**3**	254	77.6	17.7	3.9	0.8	
	**4**	75	82.7	12.0	5.3	0.0	
	**UICC**						
	**1**	300	77.7	15.3	6.0	1.0	0.4285
	**2**	37	78.4	18.9	2.7	0.0	
	**3**	91	70.3	19.8	7.7	2.2	
	**4**	75	85.3	12.0	2.7	0.0	
	**pT1**	638	78.5	15.7	5.2	0.6	0.6684
	**pT2**	135	81.5	14.1	3.7	0.7	
	**pT3‐4**	330	79.1	16.1	3.3	1.5	
	**pN0**	168	75.0	19.6	4.8	0.6	0.515
	**pN+**	26	65.4	23.1	7.7	3.8	
	**pM0**	103	72.8	21.4	5.8	0.0	0.0067
	**pM+**	95	85.3	14.7	0.0	0.0	
**Papillary renal cell carcinoma**	**ISUP**						
	**1**	32	43.8	34.4	21.9	0.0	0.0777
	**2**	126	40.5	42.1	15.1	2.4	
	**3**	73	54.8	31.5	12.3	1.4	
	**4**	6	100.0	0.0	0.0	0.0	
	**UICC**						
	**1**	88	46.6	29.5	22.7	1.1	0.5538
	**2**	13	46.2	23.1	30.8	0.0	
	**3**	5	60.0	40.0	0.0	0.0	
	**4**	10	80.0	10.0	10.0	0.0	
	**pT1**	187	42.2	38.5	17.1	2.1	0.0277
	**pT2**	45	44.4	40.0	13.3	2.2	
	**pT3‐4**	33	75.8	18.2	6.1	0.0	
	**pN0**	24	50.0	33.3	12.5	4.2	0.5105
	**pN+**	14	71.4	21.4	7.1	0.0	
	**pM0**	25	48.0	28.0	24.0	0.0	0.0751
	**pM+**	11	72.7	27.3	0.0	0.0	
**Endometrioid carcinoma of the ovary**	**pT1**	25	28.0	36.0	12.0	24.0	0.5287
	**pT2**	5	20.0	20.0	20.0	40.0	
	**pT3**	5	0.0	20.0	20.0	60.0	
	**pN0**	23	13.0	39.1	17.4	30.4	0.3563
	**pN1**	7	42.9	14.3	14.3	28.6	
**Serous carcinoma of the ovary**	**pT1**	30	46.7	26.7	6.7	20.0	0.0520
	**pT2**	42	71.4	23.8	2.4	2.4	
	**pT3**	247	66.4	24.3	5.7	3.6	
	**pN0**	80	62.5	32.5	1.3	3.8	0.0857
	**pN1**	148	72.2	19.0	4.4	4.4	
**Adenocarcinoma of the pancreas**	**pT1**	10	10	0	10	80	0.1901
	**pT2**	52	3.8	7.7	0	88.5	
	**pT3**	289	4.2	8.7	6.6	80.6	
	**pT4**	19	5.3	0	5.3	89.5	
	**G1**	12	8.3	0	8.3	83.3	0.5638
	**G2**	261	3.8	7.3	5	83.9	
	**G3**	78	3.8	11.5	7.7	76.9	
	**pN0**	75	6.7	9.3	5.3	78.7	0.6122
	**pN+**	294	3.4	7.5	5.8	83.3	
**Papillary thyroid carcinoma**	**pT1**	151	72.2	13.9	6.6	7.3	0.2017
	**pT2**	79	73.4	11.4	2.5	12.7	
	**pT3‐4**	94	61.7	14.9	10.6	12.8	
	**pN0**	90	72.2	11.1	8.9	7.8	0.0636
	**pN+**	120	55.0	18.3	10.8	15.8	
**Follicular thyroid carcinoma**	**pT1**	15	86.7	6.7	6.7	0.0	0.3861
	**pT2**	49	95.9	2.0	0.0	2.0	
	**pT3‐4**	38	94.7	5.3	0.0	0.0	
	**pN0**	29	93.1	3.4	0.0	3.4	0.8711
	**pN+**	2	100.0	0.0	0.0	0.0	
**Invasive breast cancer of no special type**	**pT1**	131	86.3	8.4	2.3	3.1	0.5365
	**pT2**	163	81.0	8.0	4.9	6.1	
	**pT3‐4**	33	75.8	9.1	9.1	6.1	
	**G1**	10	60.0	10.0	20.0	10.0	0.0129
	**G2**	176	89.2	5.7	1.7	3.4	
	**G3**	145	75.2	12.4	6.2	6.2	
	**pN0**	160	86.9	6.3	3.1	3.8	0.0269
	**pN1**	87	82.8	12.6	2.3	2.3	
	**pN2**	37	73.0	5.4	8.1	13.5	
	**pN3**	15	60.0	26.7	0.0	13.3	
**Urothelial bladder carcinoma**	**pTa G2 low**	79	17.7	12.7	5.1	64.6	0.0832
	**pTa** **G2 high**	68	25.0	16.2	10.3	48.5	
	**pTa G3**	78	16.7	25.6	12.8	44.9	
	**G2**	22	36.4	13.6	0.0	50.0	0.0367
	**G3**	409	53.8	14.9	6.8	24.4	
	**pN0**	247	53.8	13.0	6.5	26.7	0.7362
	**pN+**	155	49.7	16.8	6.5	27.1	
	**R0**	317	53.9	15.5	5.7	24.9	0.4548
	**R1**	88	46.6	13.6	8.0	31.8	
	**L0**	169	58.6	13.6	6.5	21.3	0.0212
	**L1**	147	42.2	15.0	8.2	34.7	
	**V0**	235	54.5	12.3	6.0	27.2	0.0749
	**V1**	69	44.9	20.3	13.0	21.7	

Abbreviations: BRAF, v‐RAF murine sarcoma viral oncogene homolog B1; G, grade; ISUP, International Society of Urologic Pathologists; L, lymphatic invasion status; MMR, mismatch repair; pM, pathologic status of distant metastasis; pN, pathologic lymph node status; pT, pathologic tumor stage; RAS, rat sarcoma virus; UICC, Union for International Cancer Control; V, blood vessel invasion status.

## 4 Discussion

**FIGURE 4:**
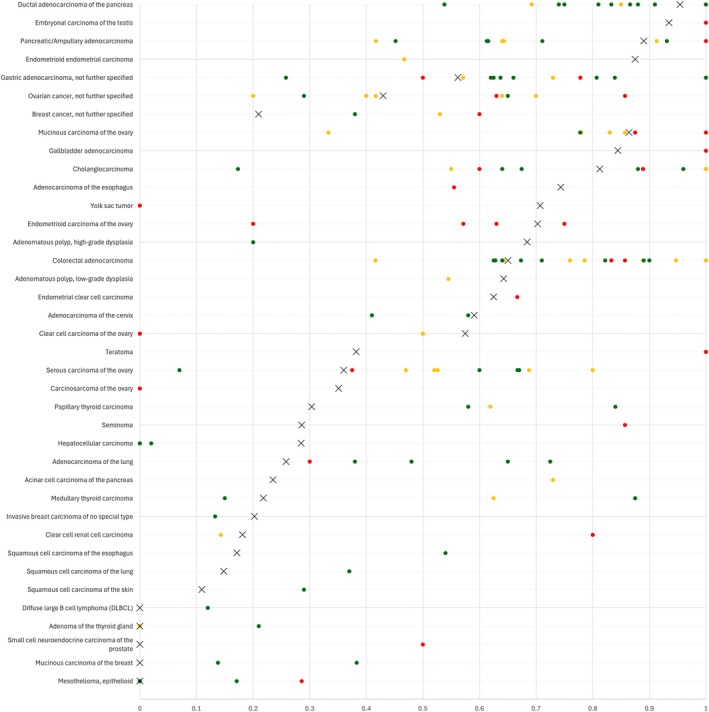
CA19‐9 protein expression in cancer (own findings vs. literature data). Graphical representation of CA19‐9 data from this study (X) compared to previous IHC studies found in the literature. The tumors were sorted according to the positivity found in this study. The colors of the dots represent the number of tumors analyzed in these studies: Red: *N* ≤ 20; yellow: *N* = 21 to 100; green: *N* > 100. For raw data and references, see Table S1.

The data from our successful analysis of 12,403 tumors from 134 different tumor categories provide a comprehensive overview on CA19‐9 expression in cancer. CA19‐9 was predominantly found in epithelial tumors, while most non‐epithelial tumors lacked CA19‐9 immunostaining. CA19‐9 positivity was most frequently seen in pancreatic ductal adenocarcinoma but it was also common in biliary and gastrointestinal adenocarcinomas, as well as in other adenocarcinomas while it was less frequent in squamous cell carcinomas and absent in tumors of the hematopoietic and lymphoid tissue and in most mesenchymal tumors. These results largely reflect the observations in normal tissue, as cancers derived from CA19‐9‐expressing normal cells were more likely to be CA19‐9‐positive than tumors derived from CA19‐9‐negative tissues. However, de novo expression or upregulation of CA19‐9 was also found in tumor entities arising from CA19‐9 negative cell types such as—for example—ovarian cancer or hepatocellular carcinoma. The CA19‐9 positivity rates described in more than 145 previous IHC studies vary considerably for many tumor entities (summarized in Figure 4), although several previous results are well in agreement with our data. For example, supporting our data, CA19‐9 positivity was earlier found in 64.3% of 14 [25] and 64% of 311 [26] colorectal cancers (our study: 65.1%), 86.6% of 155 [27] and 91% of 43 [28] ductal adenocarcinomas of the pancreas (our study: 95.4%), and in 85.7% of 14 [29] and 77.8% of 63 [30] mucinous carcinomas of the ovary (our study: 86.4%).

The ranking order of tumor entities according to their rate of CA19‐9 expression and the observation that high‐level CA19‐9 expression can occur in more than 40 different tumor entities represent some of the most important results of this study. Such an overview could not have been easily created by a compilation of the very heterogeneous methods and results which are found in the literature and often focus on individual tumor entities. In clinical practice, CA19‐9 is so far mainly used as a serological marker for pancreatic carcinomas, primarily to monitor tumor progression and therapy response. This application could be expanded to other cancer types. Several studies showed a significant correlation between CA19‐9 IHC on tissues and CA19‐9 serum levels. For example, serum CA19‐9 significantly correlated with IHC findings in intrahepatic cholangiocarcinoma [31], pulmonary adenocarcinoma [32], gastric cancer [10], and mucinous ovarian tumors [15]. The strong expression of CA19‐9 in at least a fraction of tumors from many cancer entities raises the possibility that CA19‐9 serum measurements could be a suitable biomarker for the monitoring of cancer progression and therapeutic success in CA19‐9 positive tumors of any type. Patients with tumors exhibiting a strong CA19‐9 expression might also benefit from new imaging options. In clinical studies, the radiolabeled high‐affinity antibody HuMab‐5B1 (MVT‐2163) has been successfully used to visualize CA19‐9 expressing primary tumors and metastasis [33]. Due to a significant uptake of the antibody by tumor cells, HuMab‐5B1 may also have utility for delivering therapeutic doses of radiation or anti‐cancer drugs [33]. Other approaches for utilizing CA19‐9 as a therapeutic target include human monoclonal antibodies [34, 35], tumor vaccines [36], and CA19‐9‐targeted and drug loaded nanoparticles [37].

Considering the high prevalence of CA19‐9 expression in many tumor entities, CA19‐9 IHC has little potential as a diagnostic marker for the distinction of different tumor entities. The availability of several large subsets of tumors of various entities with associated histopathological data enabled us to assess a potential prognostic role of CA19‐9 expression. That—depending on the tumor type—both high and low levels of CA19‐9 were associated with aggressive tumor features in our study is in line with a complex and cell type dependent role of CA19‐9 in cancer. The strong link between high CA19‐9 expression and unfavorable tumor features in colorectal carcinoma (CRC) is in line with data from earlier studies although most of these analyzed CA19‐9 serum levels [38, 39, 40, 41, 42, 43]. These studies described strong associations between elevated serum CA19‐9 and advanced stage [40, 41], distant metastasis [41, 43], nodal metastasis [41], BRAF‐mutations [38], and poor prognosis [39, 42, 43] of CRC. In one IHC study on 121 patients, CA19‐9 positivity was linked to nodal metastasis and poor patient outcome [44] although other authors could not confirm these findings in cohorts of 24 [11] and 78 [45] cancers. A number of mechanisms have been suggested to explain increased aggressiveness of CA19‐9 expressing cancer cells. Engle et al. described a hyperactivation of the epidermal growth factor receptor (EGFR) signaling pathway [46]. Other suggested cancer driving mechanisms of CA19‐9 include promotion of hematogenous metastasis through binding to E‐selectin [47, 48, 49], enhanced angiogenesis [47, 50], inflammation [31, 46] and a direct impact on the tumor microenvironment [51]. Of note, associations between high levels of CA19‐9 expression and dismal tumor features have earlier also been described for gastric cancer [10], thyroid cancer [52], ovarian cancer [30], and esophageal cancer [53].

We found significant associations between low CA19‐9 expression and unfavorable tumor features particularly in RCC, breast cancer, and in urothelial neoplasms. CA 19–9 IHC studies regarding these tumor entities are limited. In line with these observations, a previous study including 40 patients demonstrated a significantly lower CA19‐9 immunostaining in high‐grade urothelial carcinoma than in low‐grade tumors [54]. Other authors had also found significant associations between low CA19‐9 expression and unfavorable tumor features in endometrial [55] and pancreatic cancer [56]. The reasons for a role of CA19‐9 loss in tumor progression is unclear and studies describing molecular mechanisms that potentially can explain increased aggressiveness of tumor cells with low CA19‐9 expression are lacking. It is possible that reduced CA19‐9 expression in tumors reflects tumor cell dedifferentiation which typically parallels cancer progression. However, that CA19‐9 staining was unrelated to parameters of tumor aggressiveness in endometrial, ovarian, pancreatic and thyroidal cancer demonstrates that CA19‐9 is not a universal marker of cancer aggressiveness. It is of note, that some associations between CA19‐9 expression and tumor phenotype are not strong enough to justify CA19‐9 IHC application as a clinically relevant prognostic tool. Further evaluations in larger and more homogenously treated patient cohorts would be desirable.

Considering the large scale of our study, our assay was extensively validated according to the recommendations of the International Working Group of Antibody Validation (IWAGV) [57] by comparing our IHC findings in normal tissues with data obtained by another independent anti‐CA19‐9 antibody. To ensure as broad a range of proteins to be tested for possible cross‐reactivity, 76 different normal tissue categories were included in this analysis. These diverse tissues are likely to contain a large fraction of the proteins expressed in cells of adult humans, all of which are screened for potential cross‐reactivities of antibodies. That all normal cell findings obtained by HMV333 were confirmed by 1116‐NS‐19‐9 suggests adequate specificity of our assay.

Large‐scale studies like this are only possible with TMA technologies. All sections are stained in one experiment on the same day. IHC in a TMA format enables the simultaneous analysis of hundreds of tissue samples on a single paraffin block with maximum experimental standardization of staining and analysis [21]. There were, however, limitations to our study. The large sample size of our TMA represents a major strength of this study, as it enabled the detection of statistically significant associations between tumor genotype and phenotype, even when effect sizes were modest. However, it should be acknowledged that such weak associations may be of limited biological relevance. Although a large variety of different tumor types and subtypes were studied, the number of samples for some of the less common tumor entities was not always as high as would have been desirable. Some rare tumor types were not represented in our TMA. In addition, clinical data were not available for some tumor types to further investigate the clinical significance of CA19‐9 expression.

In summary, our data confirm that pancreatic adenocarcinoma is the cancer entity with the highest average level of CA19‐9 staining. That similarly high CA19‐9 levels can also occur in subsets of tumors from various other cancer types might suggest that many more tumors than pancreatic cancers might benefit from disease monitoring by CA19‐9 serology. Cancer type dependent associations between high or low CA19‐9 expression and aggressive tumor phenotype are in line with a complex and variable functional role of CA19‐9 in cancer cells.

## Author Contributions

**Nina Schraps:** conceptualization, methodology, investigation, formal analysis, writing – original draft, writing – review and editing. **Anne Menz:** investigation, resources, formal analysis. **Florian Viehweger:** investigation, resources, formal analysis. **Seyma Büyücek:** investigation, resources, formal analysis. **David Dum:** investigation, resources, formal analysis. **Ria Schlichter:** investigation, resources, formal analysis. **Andrea Hinsch:** investigation, resources, formal analysis. **Christoph Fraune:** investigation, resources, formal analysis. **Christian Bernreuther:** investigation, resources, formal analysis. **Martina Kluth:** investigation, resources, formal analysis. **Claudia Hube‐Magg:** investigation, resources, formal analysis. **Katharina Möller:** investigation, resources, formal analysis. **Viktor Reiswich:** investigation, resources, formal analysis. **Andreas M. Luebke:** investigation, resources, formal analysis. **Patrick Lebok:** investigation, resources, formal analysis. **Sören Weidemann:** investigation, resources, formal analysis. **Guido Sauter:** conceptualization, methodology, investigation, formal analysis, writing – original draft, writing – review and editing, supervision. **Maximilian Lennartz:** investigation, resources, formal analysis. **Frank Jacobsen:** investigation, resources, formal analysis. **Till S. Clauditz:** investigation, resources, formal analysis. **Andreas H. Marx:** investigation, resources, formal analysis. **Ronald Simon:** conceptualization, methodology, investigation, formal analysis, writing – original draft, writing – review and editing, supervision. **Stefan Steurer:** investigation, resources, formal analysis. **Baris Mercanoglu:** investigation, resources, formal analysis. **Nathaniel Melling:** investigation, resources, formal analysis. **Thilo Hackert:** investigation, resources, formal analysis. **Eike Burandt:** investigation, resources, formal analysis. **Natalia Gorbokon:** investigation, resources, formal analysis. **Sarah Minner:** investigation, resources, formal analysis. **Till Krech:** investigation, resources, formal analysis. **Florian Lutz:** investigation, resources, formal analysis. **Morton Freytag:** conceptualization, methodology, investigation, formal analysis, writing – original draft, writing – review and editing, supervision.

## Funding

The authors have nothing to report.

## Ethics Statement

The use of archived remnants of diagnostic tissues for manufacturing of TMAs and their analysis for research purposes as well as patient data analysis has been approved by local laws (HmbKHG, §12) and by the local ethics committee (Ethics commission Hamburg, WF‐049/09). All work has been carried out in compliance with the Helsinki Declaration.

## Consent

Patient consent was waived due to local laws (HmbKHG, §12,1) that permit research with anonymized diagnostic left‐over tissue samples.

## Conflicts of Interest

The CA19‐9 antibody clone HMV333 was provided by ardoci GmbH (owned by a family member of GS).

## Supporting information


**Figure S1:** IHC validation by comparison of two antibodies. The panels show a concordance of immunostaining results obtained by two independent CA19‐9 antibodies. Using HMV333, a CA19‐9 staining was seen in a subset of gastric glandular cells (A), a fraction of squamous epithelial cells in a sample from the esophagus (B), a fraction of glandular cells in the submandibular gland (C), gallbladder epithelium (D), on apical membranes of a subset of epithelial cells of the fallopian tube (E), breast epithelial cells (F), surface epithelium of the appendix (G), and in amnion cells (H). Although the staining was less intense, a comparable staining was obtained in the stomach (a), esophagus (b), submandibular gland (c), gallbladder (d), fallopian tube (e), breast (f), appendix (g), and the amnion (h) by using clone 1116‐NS‐19‐9. The images A‐H and a‐h are from consecutive tissue sections.
**Figure S2:** Graphical overview of CA19‐9 immunostaining in human tumors.

**Table S1:** List of raw data and references used to create Figure 4.
**Table S2:** Staining pattern and intensity of CA19‐9 in normal tissues in comparison to tumor tissue.

## Data Availability

The data that support the findings of this study are available from the corresponding author upon reasonable request.
