# Supplementary material for: Pattern and Clinical Significance of CA19‐9 Expression in Human Cancer: A Tissue Microarray Study on 14,966 Tumors
Source: Cancer Med. 2026 Mar 12;15(3):e71710. doi: 10.1002/cam4.71710 (PMC13093397; doi:10.1002/cam4.71710)
Supplement: Supplementary file 1 — Figure S1: IHC validation by comparison of two antibodies. The panels show a concordance of immunostaining results obtained by two independent CA19‐9 antibodies. Using HMV333, a CA19‐9 staining was seen in a subset of gastric glandular cells (A), a fraction of squamous epithelial cells in a sample from the esophagus (B), a fraction of glandular cells in the submandibular gland (C), gallbladder epithelium (D), on apical membranes of a subset of epithelial cells of the fallopian tube (E), breast epithelial cells (F), surface epithelium of the appendix (G), and in amnion cells (H). Although the staining was less intense, a comparable staining was obtained in the stomach (a), esophagus (b), submandibular gland (c), gallbladder (d), fallopian tube (e), breast (f), appendix (g), and the amnion (h) by using clone 1116‐NS‐19‐9. The images A‐H and a‐h are from consecutive tissue sections. Figure S2: Graphical overview of CA19‐9 immunostaining in human tumors. [file CAM4-15-e71710-s001.docx]

**Suppl. Figure 1. IHC validation by comparison of two antibodies.** The panels show a concordance of immunostaining results obtained by two independent CA19-9 antibodies. Using HMV333, a CA19-9 staining was seen in a subset of gastric glandular cells (**A**), a fraction of squamous epithelial cells in a sample from the esophagus (**B**), a fraction of glandular cells in the submandibular gland (**C**), gallbladder epithelium (**D**), on apical membranes of a subset of epithelial cells of the fallopian tube (**E**), breast epithelial cells (**F**), surface epithelium of the appendix (**G**), and in amnion cells (**H**). Although the staining was less intense, a comparable staining was obtained in the stomach (**a**), esophagus (**b**), submandibular gland (**c**), gallbladder (**d**), fallopian tube (**e**), breast (**f**), appendix (**g**), and the amnion (**h**) by using clone 1116-NS-19-9. The images **A-H** and **a-h** are from consecutive tissue sections.


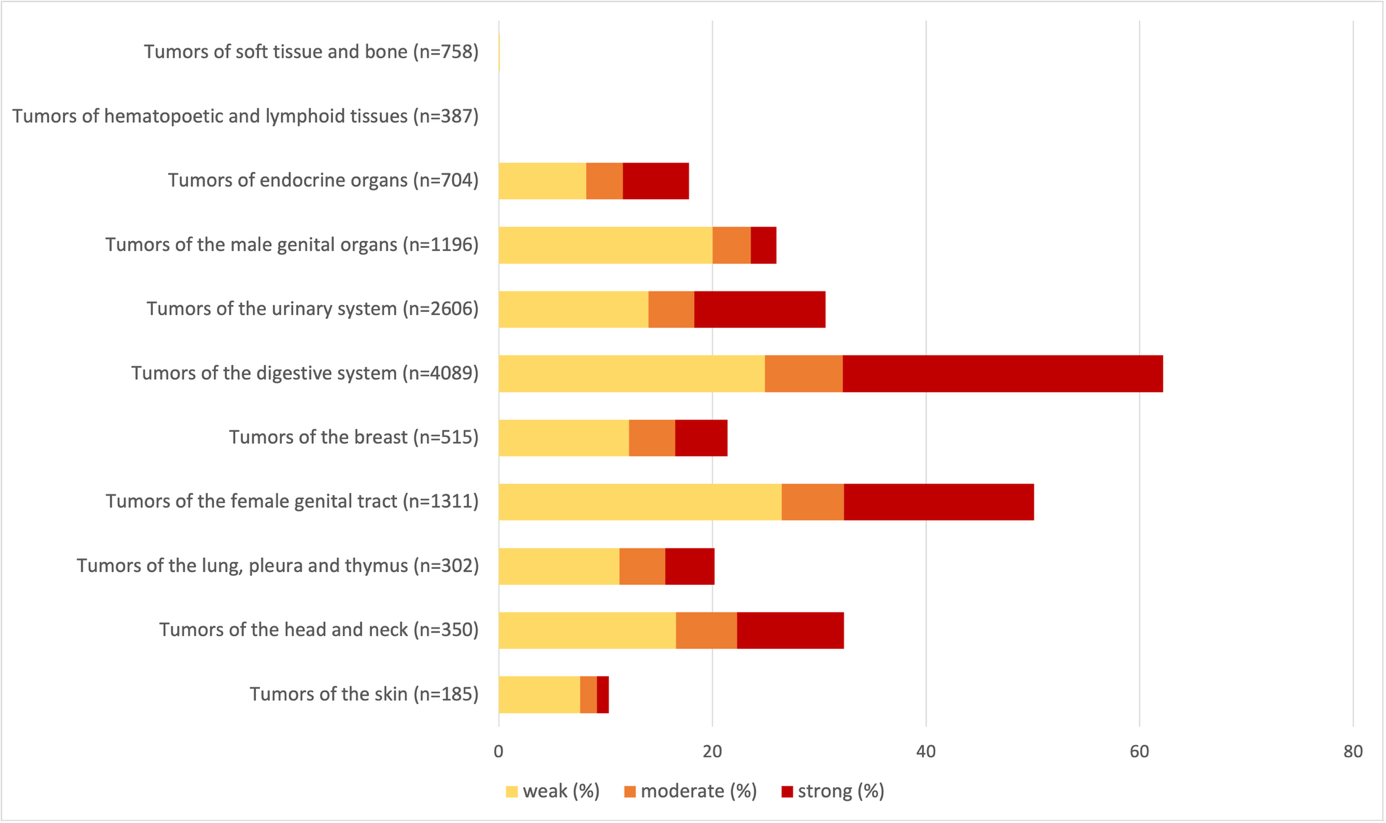


**Suppl. Figure 2. Graphical overview of CA19-9 immunostaining in human tumors.**
