# Supplementary material for: Pattern and Clinical Significance of CA19‐9 Expression in Human Cancer: A Tissue Microarray Study on 14,966 Tumors
Source: Cancer Med. 2026 Mar 12;15(3):e71710. doi: 10.1002/cam4.71710 (PMC13093397; doi:10.1002/cam4.71710)
Supplement: Supplementary file 2 — Table S1: List of raw data and references used to create Figure 4. Table S2: Staining pattern and intensity of CA19‐9 in normal tissues in comparison to tumor tissue. [file CAM4-15-e71710-s002.docx]

**Suppl Tab. 1.**

**List of raw data and references used to create Figure 4.**

| **Publication** | **PMID** | **Tumor entity** | **Number of tumors (n)** | **positivity (%)** |
| --- | --- | --- | --- | --- |
| Stelow et al. 2010 | 20182344 | Acinar cell carcinoma of the pancreas | 11 | 73% |
| Konishi et al., 1990 | 2153042 | Adenocarcinoma of the cervix | 31 | 58% |
| Nanbu et al., 1988 | 3191457 | Adenocarcinoma of the cervix | 44 | 41% |
| Sarbia et al., 1993 | 7690954 | Adenocarcinoma of the esophagus | 9 | 55% |
| Ogawa et al., 1994 | 7913734 | Adenocarcinoma of the lung | 150 | 38% |
| Kawai et al. 1993 | 8394200 | Adenocarcinoma of the lung | 102 | 65% |
| Fetsch et al. 1998 | 9570213 | Adenocarcinoma of the lung | 10 | 30% |
| Kushitani et al. 2007 | 17316414 | Adenocarcinoma of the lung | 51 | 72% |
| Sergeant et al. 2011 | 21109386 | Adenocarcinoma of the lung | 50 | 48% |
| Kamoshida et al., 2000 | 10912932 | Adenoma of the thyroid gland | 29 | 21% |
| Hoeven et al., 1998 | 9484637 | Adenoma of the thyroid gland | 18 | 0% |
| Taguchi et al. 1991 | 1716231 | Adenomatous polyp, high-grade dysplasia | 50 | 20% |
| Taguchi et al. 1991 | 1716232 | Adenomatous polyp, low-grade dysplasia | 22 | 54% |
| Fetsch et al. 1998 | 9570213 | Breast cancer, not further specified | 15 | 53% |
| Sowa et al., 1990 | 2359203 | Breast cancer, not further specified | 100 | 38% |
| Kübel et al., 1987 | 2830443 | Breast cancer, not further specified | 5 | 60% |
| Costa et al., 1992 | 1612579 | Carcinosarcoma of the ovary | 6 | 0% |
| Ohta et al., 1991 | 1655206 | Cholangiocarcinoma | 4 | 100% |
| Yamaguchi et al., 1988 | 2830725 | Cholangiocarcinoma | 82 | 67% |
| Tereda et al. 1994 | 8071522 | Cholangiocarcinoma | 14 | 100% |
| Yamauchi et al. 1993 | 8122250 | Cholangiocarcinoma | 24 | 100% |
| Tsuji et al., 2002 | 10365850 | Cholangiocarcinoma | 10 | 60% |
| Katabi el at., 2012 | 23073323 | Cholangiocarcinoma | 9 | 89% |
| Zhu et al. 2012 | 23220842 | Cholangiocarcinoma | 1 | 100% |
| Sigel et al. 2018 | 30001234 | Cholangiocarcinoma | 127 | 17% |
| Yan et al. 2019 | 31410063 | Cholangiocarcinoma | 20 | 55% |
| Zhang et al., 2022 | 34880159 | Cholangiocarcinoma | 136 | 64% |
| Gerber et al. 2022 | 35804931 | Cholangiocarcinoma | 148 | 88% |
| Gerber et al. 2022 | 35804931 | Cholangiocarcinoma | 84 | 96% |
| Tereda et al. 1992 | 1329498 | Cholangiocarcinoma | 1 | 100% |
| Helle et al., 1992 | 1594502 | Clear cell carcinoma of the ovary | 12 | 50% |
| Macdonald et al., 1988 | 3162916 | Clear cell carcinoma of the ovary | 5 | 0% |
| Nouwen et al., 1987 | 3548400 | Clear cell carcinoma of the ovary | 2 | 50% |
| Neunteufel et al. 1990 | 2646184 | Clear cell carcinoma of the ovary | 3 | 0% |
| Gilcrease et al. 1998 | 9865832 | Clear cell renal cell carcinoma | 5 | 80% |
| Ohshio et al., 1990 | 2154875 | Clear cell renal cell carcinoma | 14 | 14% |
| Kübel et al., 1987 | 2830443 | Colorectal adenocarcinoma | 6 | 83% |
| Watanabe et al. 1989 | 3042924 | Colorectal adenocarcinoma | 17 | 76% |
| Allen et al., 1987 | 3546394 | Colorectal adenocarcinoma | 14 | 89% |
| Lagendijk et al. 1998 | 9596273 | Colorectal adenocarcinoma | 46 | 89% |
| Goldstein et al., 2000 | 10981869 | Colorectal adenocarcinoma | 14 | 64% |
| Maemura et al. 2003 | 12911668 | Colorectal adenocarcinoma | 19 | 95% |
| Aoyama et al. 2007 | 17443412 | Colorectal adenocarcinoma | 1 | 100% |
| Taguchi et al. 1991 | 1716231 | Colorectal adenocarcinoma | 14 | 79% |
| Gong et al., 1985 | 2863413 | Colorectal adenocarcinoma | 45 | 82% |
| Itzkowitz et al., 1988 | 3288336 | Colorectal adenocarcinoma | 40 | 90% |
| Wiggers et al., 1988 | 3422035 | Colorectal adenocarcinoma | 311 | 64% |
| Douillard et al., 1986 | 3524803 | Colorectal adenocarcinoma | 7 | 86% |
| Ruan et al., 2004 | 15315156 | Colorectal adenocarcinoma | 20 | 100% |
| Akamine et al., 2004 | 15330211 | Colorectal adenocarcinoma | 52 | 67% |
| Imada et al. 1999 | 10228794 | Colorectal adenocarcinoma | 78 | 63% |
| Terada et al., 2013 | 23573309 | Colorectal adenocarcinoma | 12 | 100% |
| Tabuchi et al., 1988 | 3167774 | Colorectal adenocarcinoma | 83 | 62% |
| Afrem et al., 2010 | 20809023 | Colorectal adenocarcinoma | 24 | 42% |
| Nakayama et al., 1997 | 9425326 | Colorectal adenocarcinoma | 121 | 71% |
| Mohammad Foda et al. 2023 | 37654084 | Diffuse large B cell lymphoma (DLBCL) | 65 | 12% |
| Dietel et al., 1986 | 3460995 | Ductal adenocarcinoma of the pancreas | 34 | 88% |
| Kimura et al., 1992 | 1525759 | Ductal adenocarcinoma of the pancreas | 20 | 85% |
| Satomura et al., 1991 | 1678888 | Ductal adenocarcinoma of the pancreas | 42 | 83% |
| Taksaki et al., 1987 | 3320227 | Ductal adenocarcinoma of the pancreas | 38 | 81% |
| Toshkov et al., 1994 | 8071575 | Ductal adenocarcinoma of the pancreas | 31 | 74% |
| Terada et al., 1996 | 8910043 | Ductal adenocarcinoma of the pancreas | 7 | 100% |
| Hayashi et al., 2004 | 14555842 | Ductal adenocarcinoma of the pancreas | 13 | 69% |
| Ambor et al. 2006 | 16456328 | Ductal adenocarcinoma of the pancreas | 1 | 100% |
| Hamidov et al. 2011 | 21725043 | Ductal adenocarcinoma of the pancreas | 115 | 87% |
| Liu et al., 2012 | 22646265 | Ductal adenocarcinoma of the pancreas | 60 | 75% |
| Shi et al., 2014 | 24476519 | Ductal adenocarcinoma of the pancreas | 43 | 91% |
| Modi et al. 2014 | 25313783 | Ductal adenocarcinoma of the pancreas | 1 | 100% |
| Yao et al. 2016 | 27689616 | Ductal adenocarcinoma of the pancreas | 106 | 54% |
| Nicoletti et al. 2023 | 37760554 | Ductal adenocarcinoma of the pancreas | 50 | 100% |
| Tsuruta et al., 1997 | 9058515 | Embryonal carcinoma of the testis | 9 | 100% |
| Soda et al. 1998 | 9722781 | Embryonal carcinoma of the testis | 1 | 100% |
| Helle et al., 1992 | 1594502 | Endometrial clear cell carcinoma | 6 | 67% |
| Helle et al., 1992 | 1594502 | Endometrioid carcinoma of the ovary | 8 | 75% |
| Macdonald et al., 1988 | 3162916 | Endometrioid carcinoma of the ovary | 5 | 20% |
| Nouwen et al., 1987 | 3548400 | Endometrioid carcinoma of the ovary | 7 | 57% |
| Neunteufel et al. 1989 | 2646183 | Endometrioid carcinoma of the ovary | 8 | 63% |
| Helle et al., 1992 | 1594502 | Endometrioid endometrial carcinoma | 15 | 47% |
| Ono et al. 1996 | 8887046 | Gallbladder adenocarcinoma | 1 | 100% |
| Sato et al. 2009 | 19945229 | Gallbladder adenocarcinoma | 1 | 100% |
| Kato et al. 2011 | 21577374 | Gastric adenocarcinoma, not further specified | 1 | 100% |
| Terada et al., 2013 | 23573309 | Gastric adenocarcinoma, not further specified | 30 | 100% |
| Xuan et al. 1991 | 1707340 | Gastric adenocarcinoma, not further specified | 41 | 66% |
| Tabuchi et al., 1990 | 2208005 | Gastric adenocarcinoma, not further specified | 53 | 62% |
| Maeta et al., 1990 | 2342765 | Gastric adenocarcinoma, not further specified | 102 | 64% |
| Kübel et al., 1987 | 2830443 | Gastric adenocarcinoma, not further specified | 6 | 50% |
| Watanabe et al. 1988 | 3042924 | Gastric adenocarcinoma, not further specified | 11 | 73% |
| Sipponen et al., 1986 | 3464158 | Gastric adenocarcinoma, not further specified | 26 | 62% |
| Sarbia et al., 1993 | 7690955 | Gastric adenocarcinoma, not further specified | 45 | 84% |
| Sarbia et al., 1993 | 7690955 | Gastric adenocarcinoma, not further specified | 14 | 57% |
| Ikeda et al. 1995 | 7720892 | Gastric adenocarcinoma, not further specified | 52 | 81% |
| Kolodziejczyk et al. 1994 | 7954254 | Gastric adenocarcinoma, not further specified | 9 | 78% |
| Guo et al. 2011 | 22070794 | Gastric adenocarcinoma, not further specified | 1 | 100% |
| Wang et al., 2020 | 31867815 | Gastric adenocarcinoma, not further specified | 190 | 26% |
| Tsuji et al., 2002 | 10365850 | Hepatocellular carcinoma | 30 | 0% |
| Ward et al., 2010 | 20495535 | Hepatocellular carcinoma | 50 | 2% |
| Hsu et al. 2015 | 25651978 | Hepatocellular carcinoma | 90 | 0% |
| Zhang et al., 2022 | 34880159 | Hepatocellular carcinoma | 216 | 0% |
| Chu et al. 1999 | 10424790 | Invasive breast carcinoma of no special type | 81 | 13% |
| Vierbuchen et al. 1994 | 8180782 | Medullary thyroid carcinoma | 46 | 15% |
| Milman et al., 2015 | 25716629 | Medullary thyroid carcinoma | 16 | 62% |
| Vargas et al. 2020 | 32535684 | Medullary thyroid carcinoma | 78 | 87% |
| Ordónez et al., 1998 | 9777982 | Mesothelioma, epithelioid | 35 | 0% |
| Ordónez et al., 2006 | 16056246 | Mesothelioma, epithelioid | 40 | 0% |
| Kushitani et al. 2007 | 17316414 | Mesothelioma, epithelioid | 41 | 17% |
| Comin et al., 2008 | 17667536 | Mesothelioma, epithelioid | 15 | 0% |
| Sergeant et al. 2011 | 21109386 | Mesothelioma, epithelioid | 60 | 0% |
| Chu et al. 1999 | 10424790 | Mucinous carcinoma of the breast | 30 | 38% |
| Gunkel et al., 2005 | 16033095 | Mucinous carcinoma of the breast | 29 | 14% |
| Gitsch et al. 1991 | 1665684 | Mucinous carcinoma of the ovary | 14 | 86% |
| Macdonald et al., 1988 | 3162916 | Mucinous carcinoma of the ovary | 8 | 87% |
| Nouwen et al., 1987 | 3548400 | Mucinous carcinoma of the ovary | 4 | 100% |
| Lagendijk et al. 1998 | 9596273 | Mucinous carcinoma of the ovary | 18 | 78% |
| Raspollini et al., 2004 | 15354737 | Mucinous carcinoma of the ovary | 14 | 86% |
| Karaferic et al., 2009 | 20148455 | Mucinous carcinoma of the ovary | 12 | 33% |
| Kelly et al. 2010 | 20154039 | Mucinous carcinoma of the ovary | 10 | 100% |
| Gomes de Lacerda Almeida et al., 20145 | 25518016 | Mucinous carcinoma of the ovary | 63 | 78% |
| Neunteufel et al. 1989 | 2646183 | Mucinous carcinoma of the ovary | 12 | 83% |
| Nouwen et al., 1987 | 3548400 | Ovarian cancer, not further specified | 7 | 86% |
| Ruan et al., 2004 | 15315156 | Ovarian cancer, not further specified | 20 | 70% |
| Berchuck et al., 1990 | 2327461 | Ovarian cancer, not further specified | 20 | 20% |
| Breitenecker et al., 1989 | 2714933 | Ovarian cancer, not further specified | 20 | 40% |
| Fetsch et al. 1998 | 9570213 | Ovarian cancer, not further specified | 8 | 63% |
| Gitsch et al. 1991 | 1665684 | Ovarian cancer, not further specified | 12 | 42% |
| Prat et al. 1991 | 1933783 | Ovarian cancer, not further specified | 25 | 64% |
| Lagendijk et al. 1998 | 9596273 | Ovarian cancer, not further specified | 54 | 65% |
| Rosen et al., 2005 | 16061277 | Ovarian cancer, not further specified | 65 | 29% |
| Yamaguchi et al., 1991 | 2072697 | Pancreatic/Ampullary adenocarcinoma | 117 | 62% |
| Yamauchi et al. 1989 | 2815078 | Pancreatic/Ampullary adenocarcinoma | 23 | 91% |
| Kamisawa et al., 1988 | 3048082 | Pancreatic/Ampullary adenocarcinoma | 24 | 42% |
| Yamauchi et al. 1993 | 8122250 | Pancreatic/Ampullary adenocarcinoma | 29 | 93% |
| Dorandeu et al. 1997 | 9135524 | Pancreatic/Ampullary adenocarcinoma | 45 | 71% |
| Kamisawa et al. 2009 | 19214369 | Pancreatic/Ampullary adenocarcinoma | 62 | 45% |
| Tereda et al. 2012 | 22710820 | Pancreatic/Ampullary adenocarcinoma | 1 | 100% |
| Kimura et al. 1988 | 3422832 | Pancreatic/Ampullary adenocarcinoma | 14 | 64% |
| Zhou et al., 2004 | 15223956 | Pancreatic/Ampullary adenocarcinoma | 55 | 61% |
| Kimura et al., 2004 | 15368105 | Pancreatic/Ampullary adenocarcinoma | 14 | 64% |
| Hashimoto et al. 1990 | 1699752 | Papillary thyroid carcinoma | 57 | 58% |
| Kamoshida et al., 2000 | 10912932 | Papillary thyroid carcinoma | 43 | 84% |
| Hoeven et al., 1998 | 9484636 | Papillary thyroid carcinoma | 21 | 612% |
| Tsuruta et al., 1997 | 9058515 | Seminoma | 7 | 86% |
| Karaferic et al., 2009 | 20148455 | Serous carcinoma of the ovary | 43 | 7% |
| Neunteufel et al. 1989 | 2646183 | Serous carcinoma of the ovary | 21 | 52% |
| Gitsch et al., 1992 | 1376598 | Serous carcinoma of the ovary | 8 | 37% |
| Gitsch et al. 1991 | 1665684 | Serous carcinoma of the ovary | 17 | 47% |
| Macdonald et al., 1988 | 3162916 | Serous carcinoma of the ovary | 16 | 69% |
| Nouwen et al., 1987 | 3548400 | Serous carcinoma of the ovary | 19 | 53% |
| Ordónez et al., 1998 | 9777982 | Serous carcinoma of the ovary | 30 | 67% |
| Ordónez et al., 2006 | 16056246 | Serous carcinoma of the ovary | 45 | 67% |
| Comin et al., 2007 | 17667535 | Serous carcinoma of the ovary | 40 | 60% |
| Takeshima et al. 2009 | 18854271 | Serous carcinoma of the ovary | 20 | 80% |
| Okada et al. 1996 | 9008036 | Small cell neuroendocrine carcinoma of the prostate | 2 | 50% |
| Ikeda et al. 1994 | 8176946 | Squamous cell carcinoma of the esophagus | 74 | 54% |
| Ogawa et al., 1994 | 7913734 | Squamous cell carcinoma of the lung | 117 | 37% |
| Goto et al., 2015 | 26449497 | Squamous cell carcinoma of the skin | 31 | 29% |
| Macdonald et al., 1988 | 3162916 | Teratoma | 1 | 100% |
| Tsuruta et al., 1997 | 9058515 | Teratoma | 10 | 100% |
| Kitada et al. 2010 | 20549462 | Teratoma | 1 | 100% |
| Tsuruta et al., 1997 | 9058515 | Yolk sac tumor | 4 | 0% |

|  | **CA19-9**  **staining pattern of normal tissue** | **CA19-9 staining in cancer** | | | |
| --- | --- | --- | --- | --- | --- |
|  |  | **negative (%)** | **weak (%)** | **moderate**  **(%)** | **strong (%)** |
| **Esophagus** | Strong membranous staining of cells in superficial layers of the squamous epithelium | Squamous cell carcinoma of the esophagus | | | |
|  |  | 82.9 | 8.6 | 0.0 | 8.6 |
| **Stomach** | Strong staining of a subset of cells in gastric glands | Gastric adenocarcinoma, diffuse type | | | |
|  |  | 51.8 | 13.1 | 7.1 | 28.0 |
| **Colon** | weak to moderate | Adenocarcinoma of the colon | | | |
|  |  | 34.9 | 33.4 | 9.2 | 22.5 |
| **Pancreas** | Strong staining of intercalated ducts | Ductal adenocarcinoma of the pancreas | | | |
|  |  | 4.6 | 7.0 | 5.4 | 83.0 |
| **Lung** | negative | Adenocarcinoma of the lung | | | |
|  |  | 74.1 | 15.6 | 4.8 | 5.4 |
| **Liver** | Mostly absent in hepatocytes, strong staining in intrahepatic bile ducts | Hepatocellular carcinoma | | | |
|  |  | 71.5 | 10.6 | 1.8 | 16.2 |
| **Urothelium** | weak to moderate membranous staining | Urothelial carcinoma, pT2-4 G3 | | | |
|  |  | 53.8 | 15.4 | 6.5 | 24.3 |

**Suppl. Table 2 Staining pattern and intensity of CA19-9 in normal tissues in comparison to tumor tissue**
